# 1-Fluoro-4-[(*E*)-2-nitro­vin­yl]benzene

**DOI:** 10.1107/S1600536814000348

**Published:** 2014-01-11

**Authors:** S. Sreenivasa, M. S. Nanjundaswamy, K. E. Manojkumar, S. Madankumar, N. K. Lokanath, P. A. Suchetan

**Affiliations:** a Department of Studies and Research in Chemistry, Tumkur University, Tumkur, Karnataka 572 103, India; b Department of Chemistry, AVK College for Women, Davangere-2, India; c Department of Studies in Physics, University of Mysore, Manasagangotri, Mysore, India; d Department of Studies and Research in Chemistry, U.C.S., Tumkur University, Tumkur, Karnataka 572 103, India

## Abstract

The title compound, C_8_H_6_FNO_2_, is almost planar (r.m.s. deviation for the non-H atoms = 0.019 Å) and the conformation across the C=C bond is *trans*. The C and H atoms of the side chain are disordered over two sets of sites in a 0.56 (3):0.44 (3) ratio. In the crystal, mol­ecules are linked by C—H⋯O inter­actions, thus forming *C*(5) chains propagating in [001].

## Related literature   

For the biological activity of fluorinated aromatic compounds, see: Belestskaya & Cheprakov (2000 ▶). For the preparation of the title compound, see: Heck (1968 ▶).
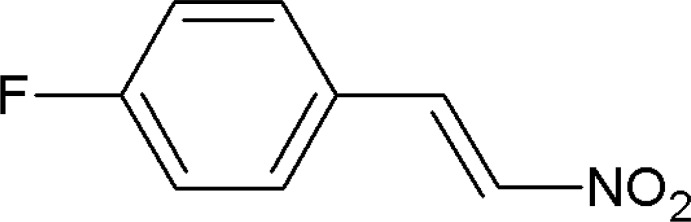


## Experimental   

### 

#### Crystal data  

C_8_H_6_FNO_2_
*M* *_r_* = 167.14
Orthorhombic, 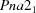

*a* = 16.0965 (14) Å
*b* = 4.8453 (4) Å
*c* = 9.5538 (9) Å
*V* = 745.12 (11) Å^3^
*Z* = 4
Cu *K*α radiation
μ = 1.08 mm^−1^
*T* = 293 K
0.33 × 0.25 × 0.19 mm

#### Data collection  

Bruker APEXII CCD diffractometer
Absorption correction: multi-scan (*SADABS*; Bruker, 2009 ▶) *T*_min_ = 0.761, *T*_max_ = 0.815
8417 measured reflections
1095 independent reflections
892 reflections with *I* > 2σ(*I*)
*R*_int_ = 0.100

#### Refinement  

*R*[*F* ^2^ > 2σ(*F* ^2^)] = 0.078
*wR*(*F* ^2^) = 0.203
*S* = 1.07
1095 reflections
129 parameters
1 restraint
H-atom parameters constrained
Δρ_max_ = 0.34 e Å^−3^
Δρ_min_ = −0.27 e Å^−3^

### 

Data collection: *APEX2* (Bruker, 2009 ▶); cell refinement: *SAINT-Plus* (Bruker, 2009 ▶); data reduction: *SAINT-Plus*; program(s) used to solve structure: *SHELXS97* (Sheldrick, 2008 ▶); program(s) used to refine structure: *SHELXL97* (Sheldrick, 2008 ▶); molecular graphics: *Mercury* (Macrae *et al.*, 2008 ▶); software used to prepare material for publication: *SHELXL97*.

## Supplementary Material

Crystal structure: contains datablock(s) I. DOI: 10.1107/S1600536814000348/hb7182sup1.cif

Structure factors: contains datablock(s) I. DOI: 10.1107/S1600536814000348/hb7182Isup2.hkl

Supporting information file. DOI: 10.1107/S1600536814000348/hb7182Isup3.cml

CCDC reference:

Additional supporting information:  crystallographic information; 3D view; checkCIF report

## Figures and Tables

**Table 1:** Hydrogen-bond geometry (Å, °)

*D*—H⋯*A*	*D*—H	H⋯*A*	*D*⋯*A*	*D*—H⋯*A*
C7*A*—H7*A*⋯O1^i^	0.93	2.57	3.408 (13)	150

Symmetry code: (i) 
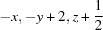
.

**Fig. 1.**
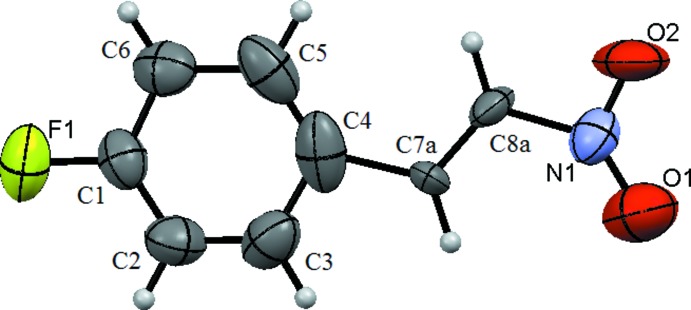
Molecular structure of the title compound, showing displacement ellipsoids at the 50% probability level. Only major components of the disordered atoms are shown.

**Fig. 2.**
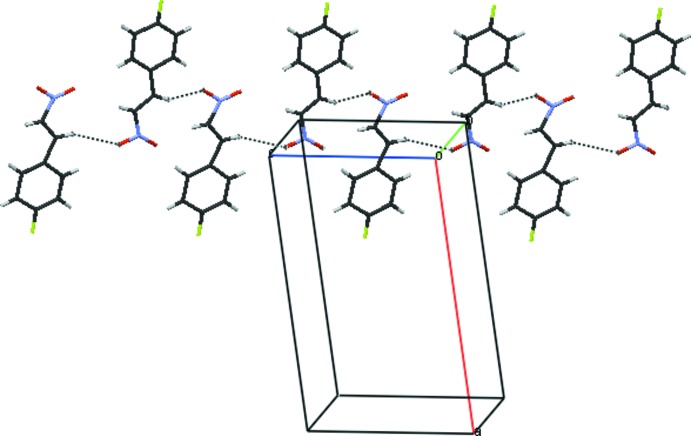
Linking of molecules into C(5) chains through C—H···O interactions.

## supplementary crystallographic information

### 1. Introduction

Fluorinated aromatic compounds exhibit different biological activities such as anti­bacterial, analgesic, anti­cancer, diuretic, anti­convulsant, insecticidal, anti­fungal and anti­viral (Belestskaya *et al.*, 2000). Further, halogenated aromatic derivatives act as a potent peptide de­formyl­ase inhibiter. As part of our studies in this area, the title compound was synthesized and its crystal structure determined.

### 2. Experimental

The title compound was synthesized by using the method reported in literature (Heck *et al.*, 1968).

### 2.1. Synthesis and crystallization

1-Bromo-4-fluoro­benzene (5.7 mmol), tetra­butyl­ammonium­bromide (11.4 mmol), cesium carbonate (17.1 mmol) and palladium acetate (5.0 mmol) were taken in N,N-di­methyl­formamide (DMF) (20 mL) and stirred for 30 minutes. To this, nitro­ethene (8.5 mmol) was added and the reaction mixture was stirred at 353K for 14 h under nitro­gen atmosphere. The confirmation of the reaction was confirmed by TLC. The organic layer was filtered and DMF was removed under vacuum. The crude product obtained was purified by column chromatography using petroleum ether and ethyl acetate (7:3) as eluent.

Colourless prisms of the title compound was obtained from slow evaporation of the solvent system: petroleum ether : ethyl acetate (8:2).

### 2.2. Refinement

The H atoms were positioned with idealized geometry using a riding model with C—H = 0.93 Å. The isotropic displacement parameters for all H atoms were set to 1.2 times Ueq(C).

### 3. Results and discussion

The title compound, C_8_H_6_FNO_2_, is almost planar (r.m.s. deviation for the non-H atoms = 0.019 Å) and the conformation across the C=C bond is *trans*. The two carbon atoms of the side chain are disordered over two sets of sites with occupancy factors 0.56 (3):0.44 (3). In the crystal structure, the molecules are linked into one another through C7A—H7A···O1 inter­actions thus forming C(5) chains.

### Crystal data

**Table:**

C_8_H_6_FNO_2_	Prism
*M**_r_* = 167.14	*D*_x_ = 1.490 Mg m^−^^3^
Orthorhombic, *P**n**a*2_1_	Melting point: 395 K
Hall symbol: P 2c -2n	Cu *K*α radiation, λ = 1.54178 Å
*a* = 16.0965 (14) Å	Cell parameters from 1213 reflections
*b* = 4.8453 (4) Å	θ = 5.5–64.6°
*c* = 9.5538 (9) Å	µ = 1.08 mm^−^^1^
*V* = 745.12 (11) Å^3^	*T* = 293 K
*Z* = 4	Prism, colourless
*F*(000) = 344	0.33 × 0.25 × 0.19 mm

### Data collection

**Table:**

Bruker APEXII CCD diffractometer	1095 independent reflections
Radiation source: fine-focus sealed tube	892 reflections with *I* > 2σ(*I*)
Graphite monochromator	*R*_int_ = 0.100
φ and ω scans	θ_max_ = 64.1°, θ_min_ = 5.5°
Absorption correction: multi-scan (*SADABS*; Bruker, 2009)	*h* = −18→18
*T*_min_ = 0.761, *T*_max_ = 0.815	*k* = −5→5
8417 measured reflections	*l* = −11→10

### Refinement

**Table:**

Refinement on *F*^2^	Secondary atom site location: difference Fourier map
Least-squares matrix: full	Hydrogen site location: inferred from neighbouring sites
*R*[*F*^2^ > 2σ(*F*^2^)] = 0.078	H-atom parameters constrained
*wR*(*F*^2^) = 0.203	*w* = 1/[σ^2^(*F*_o_^2^) + (0.1544*P*)^2^] where *P* = (*F*_o_^2^ + 2*F*_c_^2^)/3
*S* = 1.07	(Δ/σ)_max_ = 0.011
1095 reflections	Δρ_max_ = 0.34 e Å^−^^3^
129 parameters	Δρ_min_ = −0.27 e Å^−^^3^
1 restraint	Extinction correction: *SHELXL97* (Sheldrick, 2008), Fc^*^=kFc[1+0.001xFc^2^λ^3^/sin(2θ)]^-1/4^
Primary atom site location: structure-invariant direct methods	Extinction coefficient: 0.013 (4)

### Special details

**Table:**

Geometry. All s.u.'s (except the s.u. in the dihedral angle between two l.s. planes) are estimated using the full covariance matrix. The cell s.u.'s are taken into account individually in the estimation of s.u.'s in distances, angles and torsion angles; correlations between s.u.'s in cell parameters are only used when they are defined by crystal symmetry. An approximate (isotropic) treatment of cell s.u.'s is used for estimating s.u.'s involving l.s. planes.
Refinement. Refinement of *F*^2^ against ALL reflections. The weighted *R*-factor *wR* and goodness of fit *S* are based on *F*^2^, conventional *R*-factors *R* are based on *F*, with *F* set to zero for negative *F*^2^. The threshold expression of *F*^2^ > 2σ(*F*^2^) is used only for calculating *R*-factors(gt) *etc*. and is not relevant to the choice of reflections for refinement. *R*-factors based on *F*^2^ are statistically about twice as large as those based on *F*, and *R*- factors based on ALL data will be even larger.

### Fractional atomic coordinates and isotropic or equivalent isotropic displacement parameters (Å^2^)

**Table:**

	*x*	*y*	*z*	*U*_iso_*/*U*_eq_	Occ. (<1)
C1	0.22198 (14)	0.0813 (5)	0.4662 (8)	0.0574 (9)	
C2	0.1931 (4)	0.1773 (11)	0.3445 (6)	0.0639 (15)	
H2	0.2131	0.1031	0.2613	0.077*	
C3	0.1370 (4)	0.3748 (12)	0.3401 (6)	0.0701 (15)	
H3	0.1195	0.4429	0.2540	0.084*	
C4	0.10325 (17)	0.4839 (6)	0.4642 (12)	0.0776 (13)	
C5	0.1310 (3)	0.3844 (12)	0.5870 (7)	0.0758 (17)	
H5	0.1087	0.4542	0.6696	0.091*	
C6	0.1934 (3)	0.1761 (12)	0.5954 (7)	0.0671 (16)	
H6	0.2134	0.1088	0.6802	0.080*	
C7	0.0410 (7)	0.708 (2)	0.4198 (14)	0.036 (4)	0.44 (3)
H7	0.0324	0.7490	0.3259	0.043*	0.44 (3)
C8	0.0004 (8)	0.839 (2)	0.5188 (15)	0.045 (4)	0.44 (3)
H8	0.0090	0.8030	0.6134	0.053*	0.44 (3)
C7A	0.0409 (6)	0.687 (2)	0.5181 (13)	0.056 (4)	0.56 (3)
H7A	0.0311	0.7124	0.6132	0.067*	0.56 (3)
C8A	0.0010 (7)	0.828 (3)	0.4217 (14)	0.057 (4)	0.56 (3)
H8A	0.0100	0.7943	0.3270	0.068*	0.56 (3)
N1	−0.05955 (14)	1.0454 (5)	0.4692 (7)	0.0588 (8)	
F1	0.27937 (11)	−0.1214 (4)	0.4674 (6)	0.0848 (9)	
O1	−0.0826 (3)	1.1361 (12)	0.3561 (5)	0.0967 (15)	
O2	−0.0840 (3)	1.1298 (12)	0.5777 (4)	0.0886 (14)	

### Atomic displacement parameters (Å^2^)

**Table:**

	*U*^11^	*U*^22^	*U*^33^	*U*^12^	*U*^13^	*U*^23^
C1	0.0495 (14)	0.0531 (15)	0.070 (2)	0.0015 (10)	−0.001 (3)	−0.012 (3)
C2	0.081 (4)	0.061 (3)	0.050 (3)	−0.004 (3)	0.007 (3)	−0.005 (2)
C3	0.063 (3)	0.077 (4)	0.070 (4)	−0.004 (3)	−0.004 (3)	0.022 (3)
C4	0.0459 (15)	0.0480 (15)	0.139 (4)	−0.0036 (12)	0.012 (4)	0.006 (4)
C5	0.064 (3)	0.063 (3)	0.100 (4)	−0.011 (3)	0.030 (4)	−0.019 (3)
C6	0.060 (3)	0.082 (4)	0.059 (3)	−0.011 (3)	−0.004 (2)	−0.003 (3)
C7	0.039 (6)	0.044 (5)	0.025 (7)	0.011 (4)	0.012 (4)	0.001 (4)
C8	0.062 (8)	0.041 (6)	0.030 (9)	0.009 (5)	−0.013 (5)	0.008 (4)
C7A	0.066 (7)	0.067 (7)	0.035 (7)	−0.017 (5)	0.016 (4)	0.000 (4)
C8A	0.058 (6)	0.085 (8)	0.028 (7)	−0.003 (6)	−0.007 (4)	0.004 (4)
N1	0.0559 (13)	0.0616 (13)	0.0589 (18)	0.0046 (10)	−0.015 (2)	0.003 (3)
F1	0.0633 (11)	0.0673 (12)	0.124 (2)	0.0142 (7)	−0.004 (2)	0.018 (3)
O1	0.103 (4)	0.118 (3)	0.069 (3)	−0.007 (3)	−0.003 (3)	0.014 (3)
O2	0.104 (3)	0.129 (4)	0.033 (2)	−0.003 (3)	0.007 (3)	0.005 (2)

### Geometric parameters (Å, º)

**Table:**

C1—C2	1.335 (10)	C6—H6	0.9300
C1—F1	1.348 (3)	C7—C8	1.31 (2)
C1—C6	1.395 (9)	C7—H7	0.9300
C2—C3	1.317 (8)	C8—N1	1.470 (13)
C2—H2	0.9300	C8—H8	0.9300
C3—C4	1.407 (12)	C7A—C8A	1.31 (2)
C3—H3	0.9300	C7A—H7A	0.9300
C4—C5	1.345 (12)	C8A—N1	1.506 (16)
C4—C7A	1.499 (13)	C8A—H8A	0.9300
C4—C7	1.536 (14)	N1—O2	1.182 (8)
C5—C6	1.426 (9)	N1—O1	1.224 (8)
C5—H5	0.9300		
			
C2—C1—F1	119.9 (6)	C5—C6—H6	122.7
C2—C1—C6	122.8 (3)	C8—C7—C4	117.8 (14)
F1—C1—C6	117.3 (6)	C8—C7—H7	121.1
C3—C2—C1	121.3 (5)	C4—C7—H7	121.1
C3—C2—H2	119.4	C7—C8—N1	115.1 (13)
C1—C2—H2	119.4	C7—C8—H8	122.5
C2—C3—C4	120.7 (5)	N1—C8—H8	122.5
C2—C3—H3	119.6	C8A—C7A—C4	115.3 (13)
C4—C3—H3	119.6	C8A—C7A—H7A	122.3
C5—C4—C3	118.2 (3)	C4—C7A—H7A	122.3
C5—C4—C7A	99.1 (9)	C7A—C8A—N1	117.9 (13)
C3—C4—C7A	142.7 (10)	C7A—C8A—H8A	121.0
C5—C4—C7	135.3 (9)	N1—C8A—H8A	121.0
C3—C4—C7	106.5 (9)	O2—N1—O1	123.3 (3)
C4—C5—C6	122.5 (5)	O2—N1—C8	99.9 (8)
C4—C5—H5	118.8	O1—N1—C8	136.7 (8)
C6—C5—H5	118.8	O2—N1—C8A	136.2 (7)
C1—C6—C5	114.5 (5)	O1—N1—C8A	100.5 (7)
C1—C6—H6	122.7		
			
F1—C1—C2—C3	179.9 (5)	C3—C4—C7—C8	179.3 (7)
C6—C1—C2—C3	2.0 (6)	C7A—C4—C7—C8	2.0 (7)
C1—C2—C3—C4	−2.5 (9)	C4—C7—C8—N1	−178.6 (6)
C2—C3—C4—C5	1.2 (6)	C5—C4—C7A—C8A	179.5 (7)
C2—C3—C4—C7A	−177.6 (6)	C3—C4—C7A—C8A	−1.5 (12)
C2—C3—C4—C7	179.8 (6)	C7—C4—C7A—C8A	2.8 (7)
C3—C4—C5—C6	0.5 (6)	C4—C7A—C8A—N1	−177.2 (5)
C7A—C4—C5—C6	179.8 (6)	C7—C8—N1—O2	−178.5 (8)
C7—C4—C5—C6	−177.5 (6)	C7—C8—N1—O1	−1.1 (13)
C2—C1—C6—C5	−0.2 (5)	C7—C8—N1—C8A	3.3 (7)
F1—C1—C6—C5	−178.2 (3)	C7A—C8A—N1—O2	−1.2 (12)
C4—C5—C6—C1	−1.0 (8)	C7A—C8A—N1—O1	178.4 (7)
C5—C4—C7—C8	−2.5 (11)	C7A—C8A—N1—C8	1.4 (7)

### Hydrogen-bond geometry (Å, º)

**Table:**

*D*—H···*A*	*D*—H	H···*A*	*D*···*A*	*D*—H···*A*
C7*A*—H7*A*···O1^i^	0.93	2.57	3.408 (13)	150

Symmetry code: (i) −*x*, −*y*+2, *z*+1/2.
